# Limited Immune-Mediated Efficacy of Anti-PD-L1/VEGF in EGFR-TKI-Naïve *Egfr*-Mutant Lung Cancer with Non-Inflamed Tumor Microenvironment

**DOI:** 10.3390/curroncol33060315

**Published:** 2026-05-27

**Authors:** Atsuko Hirabae, Tadahiro Kuribayashi, Shuta Tomida, Sachi Okawa, Takamasa Nakasuka, Kazuya Nishii, Jun Nishimura, Go Makimoto, Kiichiro Ninomiya, Hisao Higo, Kammei Rai, Eiki Ichihara, Katsuyuki Hotta, Masamichi Sugimoto, Yosuke Togashi, Yoshinobu Maeda, Katsuyuki Kiura, Kadoaki Ohashi

**Affiliations:** 1 Department of Hematology, Oncology and Respiratory Medicine, Graduate School of Medicine, Dentistry and Pharmaceutical Sciences, Okayama University, Okayama 700-8558, Japan; 2 Center for Comprehensive Genomic Medicine, Okayama University Hospital, Okayama 700-8558, Japan; 3 Department of Respiratory Medicine, Okayama University Hospital, Okayama 700-8558, Japan; 4 Center for Innovative Clinical Medicine, Okayama University Hospital, Okayama 700-8558, Japan; 5 Center for Clinical Oncology, Okayama University Hospital, Okayama 700-8558, Japan; 6 Product Research Department, Kamakura Research Laboratories, Chugai Pharmaceutical Co., Ltd., Kamakura 247-8530, Japan

**Keywords:** EGFR mutation, non-small cell lung cancer, syngeneic mouse model, antitumor immunity, non-inflamed tumor, VEGF, PD-L1

## Abstract

Immune checkpoint inhibitors (ICIs) show limited efficacy in epidermal growth factor receptor (*EGFR*)-mutant lung adenocarcinoma due to its non-inflamed tumor microenvironment (TME). While quadruple therapy combining chemotherapy, anti-programmed death-ligand 1 (PD-L1), and anti-vascular endothelial growth factor (VEGF) antibodies has shown inconsistent results in EGFR-tyrosine kinase inhibitor (TKI)-pretreated patients, whether anti-VEGF therapy can modulate the intrinsic non-inflamed TME remains unknown. We employed an EGFR-TKI-naïve syngeneic *Egfr*-mutant mouse model and evaluated effects of anti-VEGF, anti-PD-L1, carboplatin, and paclitaxel as monotherapies and combinations, with TME analysis via immunohistochemistry (IHC), flow cytometry, and RNA sequencing. Anti-PD-L1 showed no antitumor effect, and adding anti-VEGF failed to convert the TME to an inflamed status. Although paclitaxel—but not carboplatin—combined with low-dose anti-VEGF inhibited tumor growth, adding anti-PD-L1 provided no benefit, indicating the anti-VEGF-A antibody evaluated here has a limited role in sensitizing tumors to anti-PD-L1 regardless of chemotherapy. CD8^+^ T-cell depletion did not attenuate the effect of paclitaxel plus low-dose anti-VEGF, and IHC and RNA sequencing revealed increased natural killer cell infiltration, suggesting a CD8^+^ T-cell-independent, innate immune mechanism. These findings provide preclinical evidence that the evaluated anti-VEGF has limited immunomodulatory activity in EGFR-TKI-naïve *Egfr*-mutant tumors with a non-inflamed TME, and suggest immunotherapeutic strategies beyond CD8^+^ T-cell-mediated immunity warrant investigation.

## Simple Summary

Lung cancers with mutations in the epidermal growth factor receptor gene often respond poorly to immune checkpoint inhibitors, largely because the tumor environment lacks sufficient immune activity. Some clinical trials have tested whether adding a drug targeting tumor blood vessels to immunotherapy and chemotherapy could overcome this limitation, but results have been inconsistent. A key question is whether such drugs can activate the immune system in tumors not yet exposed to targeted therapies. We investigated this in a mouse model mimicking the poorly immunogenic environment of human *EGFR*-mutant lung cancer before targeted therapy and found that the anti-VEGF drug we tested did not activate the immune system or improve checkpoint inhibitor effectiveness. However, combining paclitaxel with this drug suppressed tumor growth through a mechanism that did not rely on T cells, with increased natural killer cell infiltration. These findings suggest that future immunotherapy strategies may need to engage immune pathways beyond T-cell responses.

## 1. Introduction

Immune checkpoint inhibitors (ICIs) are a standard treatment for advanced cancer, providing significant survival benefits across various malignancies [1], including lung cancer, the leading cause of cancer-related mortality worldwide [2]. However, their efficacy is notably limited in patients with *EGFR*-mutant non-small cell lung cancers (NSCLCs) [3,4]. This attenuated response is generally attributed to a lower tumor mutational burden (TMB) and a non-inflamed tumor microenvironment (TME), characterized by a paucity of CD8^+^ T-cell infiltration relative to *EGFR*-wild type NSCLCs [5,6]. Given that *EGFR* mutations account for up to 50–60% of driver mutations in lung adenocarcinoma associated with never-smoking status [7], there is a critical unmet need to develop more effective immunotherapy strategies for this specific patient population.

Vascular endothelial growth factor (VEGF) plays a pivotal role in modulating the TME, promoting both tumor angiogenesis and immunosuppression [8]. Mechanistically, VEGF is known to impair effector T-cell function and to recruit immunosuppressive cells, such as regulatory T-cells (Tregs) and M2 macrophages [9]. Building on this rationale, combination therapies integrating ICIs with VEGF/VEGF receptor (VEGFR) inhibitors have been developed, demonstrating improved survival outcomes in highly vascularized cancers, such as renal cell carcinoma and hepatocellular carcinoma [10,11,12].

While the combination of atezolizumab (anti-PD-L1 antibody) and bevacizumab (anti-VEGF antibody) has yielded promising results in *EGFR*-wild-type NSCLC with high TMB or PD-L1 expression [13,14], the potential benefit of adding anti-VEGF therapy to ICI monotherapy in *EGFR*-mutant lung adenocarcinoma remains controversial. Furthermore, the efficacy of quadruple therapy comprising platinum-doublet chemotherapy plus ICI (Chemo-IO) and an anti-VEGF antibody has yielded inconsistent outcomes. Exploratory analyses of the IMpower150 trial and the WJOG11218L study, both involving patients pretreated with EGFR-TKIs, suggested an overall survival benefit with quadruple therapy compared with chemotherapy plus anti-VEGF antibody or Chemo-IO alone [15,16]. In contrast, other studies, such as the IMpower151 study, failed to demonstrate significant survival prolongation with quadruple therapy in this subgroup [17]. Consequently, whether anti-VEGF antibodies effectively potentiate antitumor immunity in *EGFR*-mutant lung cancer remains a subject of debate.

A major limitation when addressing this question is the scarcity of relevant preclinical in vivo models for studying antitumor immunity specifically in *EGFR*-mutant lung cancer. To date, no studies have directly evaluated the immunostimulatory potential of anti-VEGF antibodies in this specific setting. To address this gap, we previously developed a genetically engineered mouse model (GEMM) of spontaneous *EGFR*-mutant lung adenocarcinoma arising from type II pneumocytes [18,19]. Subsequently, we established a corresponding syngeneic model via the subcutaneous transplantation of tumor cells derived from this GEMM into immunocompetent C57BL/6 mice [20]. This syngeneic model recapitulates key characteristics of human *EGFR*-mutant lung cancer, including minimal PD-L1 expression and a non-inflamed TME [21]. In the present study, we focused specifically on the EGFR-TKI-naïve setting to isolate the intrinsic immunological properties of *Egfr*-mutant tumors from potential confounding effects of prior TKI-induced TME remodeling, and to directly address whether anti-VEGF therapy can modulate the non-inflamed TME inherent to *EGFR*-mutant lung cancer. Leveraging this model to assess antitumor immunity, we aimed to investigate the efficacy of anti-VEGF antibody in combination with anti-PD-L1 antibody or cytotoxic chemotherapy in *Egfr*-mutant lung cancer.

## 2. Materials and Methods

### 2.1. Syngeneic Egfr-Mutant Lung Cancer Mouse Model

Experiments were conducted with the approval of the Animal Care and Use Committee, Okayama University (Okayama, Japan, OKU 2020142) and in accordance with the ARRIVE guidelines. Female C57BL/6J mice (7 weeks old) purchased from Charles River Laboratories Japan (Yokohama, Japan) were maintained under pathogen-free conditions in the animal facility at Okayama University.

Subcutaneous *Egfr*-mutant lung tumors were minced and dissociated into single-cell suspensions using a gentleMACS Octo Dissociator with Heaters (Miltenyi Biotec, Bergisch Gladbach, Germany) according to the protocol of a mouse Tumor Dissociation Kit (Miltenyi Biotec, #130-096-730). A Red Blood Cell Lysis Solution (Miltenyi Biotec, #130-094-183) was used to remove erythrocytes from the suspensions. C57BL/6J mice were inoculated subcutaneously with 7–8 × 10^5^ *Egfr*-mutant lung cancer cells suspended in 50% Matrigel solution (Corning, New York, NY, USA, #356237) or 2 × 10^5^ MC-38 mouse colon cancer cells (Kerafast, Boston, MA, USA, #KER-ENH204).

When the average tumor volume reached approximately 150 mm^3^, *Egfr*-mutant lung tumor-bearing mice were randomized into control and treatment groups. Mice were administered phosphate-buffered saline (PBS), carboplatin (30 mg/kg, 1, 2, or 4–5 times a week), or paclitaxel (10 or 20 mg/kg, once a week) anticancer agents from EVERLTH (Hiroshima, Japan), via intraperitoneal injection, erlotinib (30 mg/kg, 5 times a week orally) from Funakoshi (Tokyo, Japan), or osimertinib (15 mg/kg, 5 times a week orally) from EVERLTH. Anti-VEGFA antibody (B20-4.1.1.-PHAGE, 1 or 5 mg/kg [22,23]) and anti-PD-L1 antibody (clone 6E11, 5 mg/kg) were provided by Genentech (South San Francisco, CA, USA) under the material transfer agreement and administered intraperitoneally to mice twice weekly. In the MC38 mouse model, mice were randomized into a control group and a treatment group on the day of inoculation and treated with PBS or anti-PD-L1 antibody as described above. For CD8 depletion, mice were administered PBS or anti-CD8α antibody (clone 2.43, 10 mg/kg; BioXcell, Lebanon, NH, USA) intraperitoneally on days 1–3, 8, and 12.

The tumor size was measured using digital calipers, and the tumor volume was calculated as volume (mm^3^) = length × width^2^/2. Time-to-event curves were generated by the Kaplan–Meier method, with the event defined as a tumor volume of 1500 mm^3^; this threshold was set below the institutional humane endpoint of 2000 mm^3^ to provide an objective surrogate of local tumor progression. Mice were euthanized by cervical dislocation under deep isoflurane anesthesia when tumor volume exceeded 2000 mm^3^ or when they were moribund.

### 2.2. Immunohistochemistry Analysis (Ihc)

Subcutaneous tumors were excised on day 5 (carboplatin or paclitaxel monotherapy) or day 8 (all other therapies) post-treatment. Tissues were fixed in formalin, embedded in paraffin, and sectioned at a thickness of 4 μm. Slides were deparaffinized and rehydrated, and antigen retrieval was performed by boiling in 1 mM EDTA (pH 8.0) or Tris-EDTA Antigen Retrieval Buffer (Proteintech, Rosemont, IL, USA; PR30002) using a Pascal pressure chamber (Dako, Glostrup, Denmark, #S2800). After blocking with 0.3% H_2_O_2_ in methanol and normal goat serum, the slides were incubated with primary antibodies against CD8α (1:2000, EPR21769), and NCR1 (1:250, EPR23097-35) from Abcam (Cambridge, UK), or against PD-L1 (1:200, D5V3B), CD31 (1:100, D8V9E), Foxp3 (1:100, D608R), CD206 (1:400, E6T5J), Granzyme B (1:100, E5V2L), αSMA (1:600, D4K9N), and NK1.1 (1:800, E6Y9G) from Cell Signaling Technology (Danvers, MA, USA). The secondary antibodies used were EnVision+ System-HRP Labelled Polymer Anti-Rabbit (Dako, #K4002) or SignalStain Boost IHC Detection Reagent (HRP, Rabbit; Cell Signaling Technology, #8114), according to the manufacturer’s instructions. Liquid DAB+ Substrate Chromogen System (Dako, #K3468) was used as the chromogen, and tissue sections were counterstained with Mayer’s hematoxylin.

The images were acquired using a BZ-X700 microscope (Keyence, Osaka, Japan) and quantified with the image stitching function of BZ-X Analyzer software (Version 1.4.10; Keyence).

### 2.3. Flow Cytometry (Fcm)

*Egfr*-mutant lung tumors were harvested on day 5 (carboplatin or paclitaxel monotherapy) or day 8 (all other therapies) and dissociated into single-cell suspensions as described above. Cells were washed and incubated with monoclonal antibodies for 30 min at 4 °C in staining buffer (2 mM EDTA, 0.5% BSA in PBS). Cell-surface markers were stained with fluorochrome-conjugated antibodies against CD4 (efluor450, GK1.5), CD8α (FITC, 53-6.7), CD3 (PerCP-eFluor710, 17A2), and PD-1 (PE, RMP1-30) from Thermo Fisher Scientific (Waltham, MA, USA), or against CD4 (FITC, GK1.5, or PE/Cy7, GK1.5) and CD8α (APC/Cy7, 53-6.7) from BioLegend (San Diego, CA, USA). DAPI (#422801) or the Zombie NIR Fixable Viability Kit (#423105) purchased from BioLegend were used for dead cell exclusion. Samples were run on a MACSQuant flow cytometer (Miltenyi Biotec) and the data were analyzed using FlowJo software (Version 10.6.2.0, TreeStar, Ashland, OR, USA). For the validation of systemic CD8^+^ T-cell depletion, spleens were harvested on days 4, 10, and 18 after the initiation of antibody administration, processed into single-cell suspensions, and stained using the same protocol.

### 2.4. RNA Expression Profiling Using Next-Generation Sequencing

Subcutaneous *Egfr*-mutant lung tumors on day 8 post-treatment were snap-frozen in liquid nitrogen immediately after harvesting and subsequently stored at −80 °C. RNA was extracted using the RNeasy Mini Kit or AllPrep DNA/RNA Mini Kit (Qiagen, Venlo, Netherlands). Total RNA concentration and quality were assessed using Qubit (Thermo Fisher Scientific) and RNA ScreenTape Analysis (Agilent, Santa Clara, CA, USA). Ribosomal RNA was depleted from total RNA using the NEBNext rRNA Depletion Kit v2 (Human/Mouse/Rat; cat. no. E7405; New England Biolabs, Tokyo, Japan Inc.), and cDNA libraries were subsequently prepared with the MGIEasy RNA Directional Library Prep Set (MGI Tech, Tokyo, Japan). The libraries were sequenced in 150 bp paired-end mode on a DNBSEQ-G400RS sequencer with the DNBSEQ-G400RS High-throughput Sequencing Set (cat. no. FCL PE150; MGI Tech, Tokyo, Japan). Sequencing reads were aligned to the mouse reference genome (GRCm38.p6) using CLC Genomics Workbench (Version 26.0.2, CLC Bio, Aarhus, Denmark), and gene expression levels for protein-coding genes were quantified as transcripts per million (TPM), calculated by dividing read counts by gene length in kilobases (RPK) and then by the per-million scaling factor derived from the sum of RPKs. The murine Microenvironment Cell Population (mMCP)-counter method was used to estimate the proportion of tumor-infiltrating immune cells in the tumor samples [24].

### 2.5. Statistical Analysis

Statistical analyses were performed using GraphPad Prism 9 software (GraphPad Software, San Diego, CA, USA). Unpaired Student’s *t*-test was used to compare two experimental groups. One-way ANOVA with Tukey’s test was used for comparing more than two groups. Survival curves were estimated using Kaplan–Meier methods and compared using the log-rank test. Data are presented as the mean ± SEM. *p*–values < 0.05 were considered statistically significant.

## 3. Results

### 3.1. Vegf Inhibition Does Not Sensitize Egfr-Mutant Tumors to Anti-Pd-L1 Therapy

**Figure 1:**
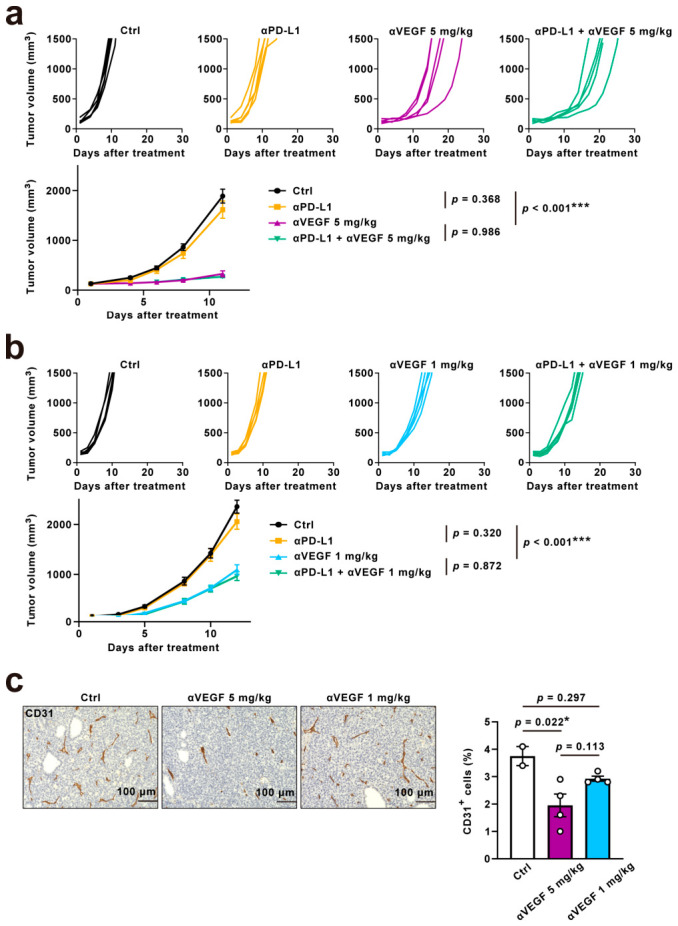
Effect of VEGF inhibition on sensitivity to anti-PD-L1 therapy in *Egfr*-mutant tumors. (**a**,**b**) Individual tumor growth curves (**top**) and mean tumor volumes (**bottom**) in the *Egfr*-mutant syngeneic mouse model treated with anti-PD-L1 (5 mg/kg), anti-VEGF (5 mg/kg or 1 mg/kg), or the combination. *n* = 5 mice per group. (**c**) Representative images of immunohistochemistry for CD31 staining in *Egfr*-mutant lung tumors according to the dose of anti-VEGF (**left**) and quantification of the percentage area of CD31^+^ cells (**right**). *n* = 2–4 samples per group. Data are presented as mean ± S.E.M.; statistical analyses were performed using one-way ANOVA with Tukey’s post hoc test; *, *p* < 0.05; ***, *p* < 0.001.

We first assessed the efficacy of anti-PD-L1 monotherapy in our syngeneic mouse model of *Egfr*-mutant lung cancer, which is sensitive to erlotinib or osimertinib (Supplementary Figure S1a). Consistent with prior reports of limited anti-PD-1 antibody efficacy in this model [21,25], anti-PD-L1 monotherapy showed no significant antitumor effect (Figure 1a). To distinguish between potential technical issues and intrinsic tumor characteristics, we first verified the in vivo functionality of the anti-PD-L1 in the highly immunogenic MC38 colorectal cancer model, in which it significantly suppressed tumor growth (Supplementary Figure S1b) [26]. We next examined baseline PD-L1 expression in untreated *Egfr*-mutant tumors by immunohistochemistry, which revealed minimal to no detectable PD-L1 staining in the tumor cells, in contrast to the strong reactivity in the thymus used as a positive control (Supplementary Figure S1c). These results suggest our model is refractory to ICI monotherapy, consistent with insufficient target expression in a non-inflamed TME.

We then evaluated the antitumor effect of anti-VEGF (5 mg/kg, the standard active dose [22]) in the model. Notably, anti-VEGF monotherapy significantly reduced tumor growth compared to vehicle control (Figure 1a). However, combining anti-PD-L1 with a 5 mg/kg dose of anti-VEGF did not induce superior efficacy compared to anti-VEGF alone (Figure 1a). Given reports suggesting that high-dose anti-angiogenic therapy may impair immunotherapy by inducing excessive vascular disruption and hypoxia [27], we tested a lower dose of anti-VEGF (1 mg/kg, one-fifth of the standard dose) based on the vascular normalization window concept [23]. This low dose also exhibited antitumor activity (Figure 1b) but did not significantly reduce the percentage area of CD31^+^ cells compared to the control group (Figure 1c). In contrast, the higher dose (5 mg/kg) significantly decreased the percentage area of CD31^+^ cells, indicating a stronger anti-angiogenic effect (Figure 1c). We subsequently investigated the efficacy of combining anti-PD-L1 with low-dose anti-VEGF (1 mg/kg), but this combination also failed to induce a superior antitumor activity compared to anti-VEGF monotherapy (Figure 1b).

To assess the TME, we performed IHC analysis, which revealed that anti-PD-L1 monotherapy increased CD8^+^ cells predominantly in the peritumoral stroma (*p* = 0.030), with a significant but numerically small increase within the intratumoral nests (*p* = 0.027) compared to the control (Supplementary Figure S2a,b). Anti-VEGF monotherapy and the combination of anti-PD-L1 and anti-VEGF did not significantly alter CD8^+^ cell counts (Supplementary Figure S2a,b). In addition, granzyme B expression, which was scarcely detectable in the control tumors, remained unchanged in those treated with either monotherapy or the combination therapy (Supplementary Figure S2a,c), suggesting that cytotoxic T lymphocytes (CTLs) were not activated. Neither Foxp3^+^ cells, a marker of regulatory T cells (Tregs), nor CD206^+^ cells, a marker of immunosuppressive M2 macrophages, showed a significant decrease in the tumors treated with combination therapy compared to those treated with either monotherapy or control (Supplementary Figure S2a,c). These results indicate that anti-VEGF therapy failed to sensitize *Egfr*-mutant tumors to anti-PD-L1 or to convert the TME from a non-inflamed to inflamed status.

### 3.2. Paclitaxel, Not Carboplatin, Increases Cd8^+^ T Cell Infiltration in Egfr-Mutant Tumors

**Figure 2:**
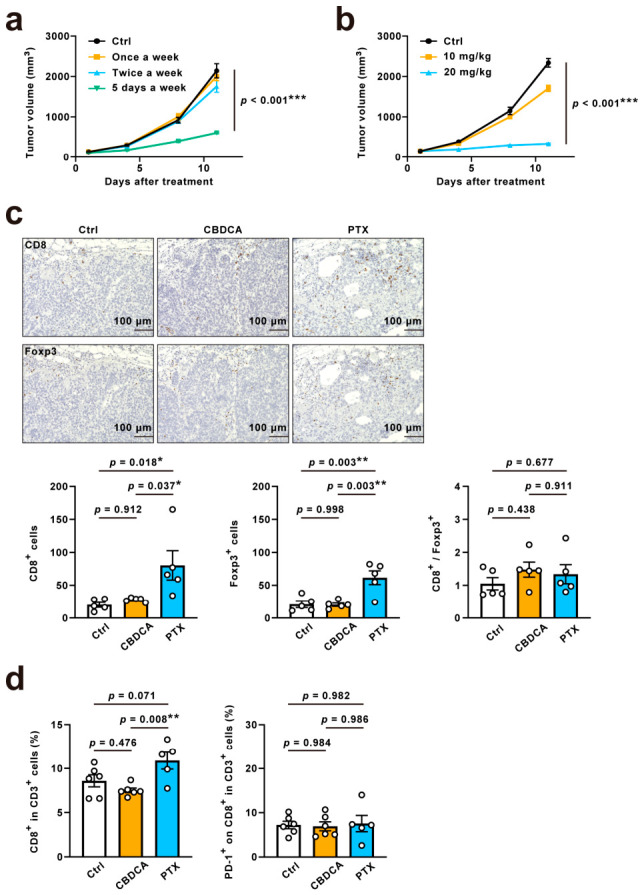
Impact of paclitaxel on the tumor microenvironment of *Egfr*-mutant tumors. (**a**,**b**) Dose-dependent antitumor effects of carboplatin (once or twice a week: *n* = 6 mice; 5 days a week: *n* = 4 mice) (**a**) or paclitaxel (*n* = 6 mice) (**b**) in the *Egfr*-mutant syngeneic mouse model. Control group (*n* = 6–12 mice). (**c**) Immunohistochemical comparison of CD8^+^ cells and Foxp3^+^ cells infiltrating *Egfr*-mutant lung tumors during carboplatin or paclitaxel administration (**top**: representative images; **bottom**: quantification of positive cell counts). *n* = 5 samples per group. (**d**) Ratio of CD8^+^ T cells among CD3^+^ cells and expression of PD-1 on CD8^+^ T cells in *Egfr*-mutant lung tumors treated with carboplatin or paclitaxel. *n* = 5–6 samples per group. Data are presented as mean ± S.E.M.; statistical analyses were performed using one-way ANOVA with Tukey’s post hoc test; *, *p* < 0.05; **, *p* < 0.01; ***, *p* < 0.001.

Given the subset analysis of the IMpower150 trial, which suggested the overall survival benefit with the quadruple combination therapy of carboplatin, paclitaxel, atezolizumab, and bevacizumab [15], we investigated the impact of carboplatin and paclitaxel on tumor immunity in our *Egfr*-mutant lung cancer mouse model. Both agents exhibited dose-dependent antitumor activity in the *Egfr*-mutant lung tumor model (Figure 2a,b).

IHC analysis showed a significant increase in the number of CD8^+^ or Foxp3^+^ cells in paclitaxel-treated tumors compared to carboplatin-treated or vehicle control tumors. The CD8^+^/Foxp3^+^ ratio, however, did not significantly differ across groups (Figure 2c), indicating a proportional recruitment of both effector and regulatory lymphocyte populations. FCM analysis showed a similar trend, with a significant increase in CD8^+^ T lymphocytes in paclitaxel-treated tumors compared to those treated with carboplatin, and a numerical but non-significant increase compared to vehicle control (Figure 2d and Figure S3). To further investigate the role of CD8^+^ T cells in the antitumor effect, we examined PD-1 expression on tumor-infiltrating lymphocytes (TILs), as a marker of CD8^+^ T cell clonal expansion and tumor antigen recognition [28]. However, PD-1 expression on CD8^+^ T cells did not significantly increase in tumors treated with paclitaxel or carboplatin compared to vehicle control (Figure 2d and Figure S3), suggesting that although paclitaxel promotes T-cell infiltration, it does not elicit functional activation of these cells. Whether this paclitaxel-induced lymphocyte infiltration functionally contributed to antitumor immunity in *Egfr*-mutant tumors remained to be determined.

### 3.3. Low-Dose Anti-Vegf, but Not Anti-Pd-L1, Enhances the Antitumor Activity of Paclitaxel in Egfr-Mutant Tumors

**Figure 3:**
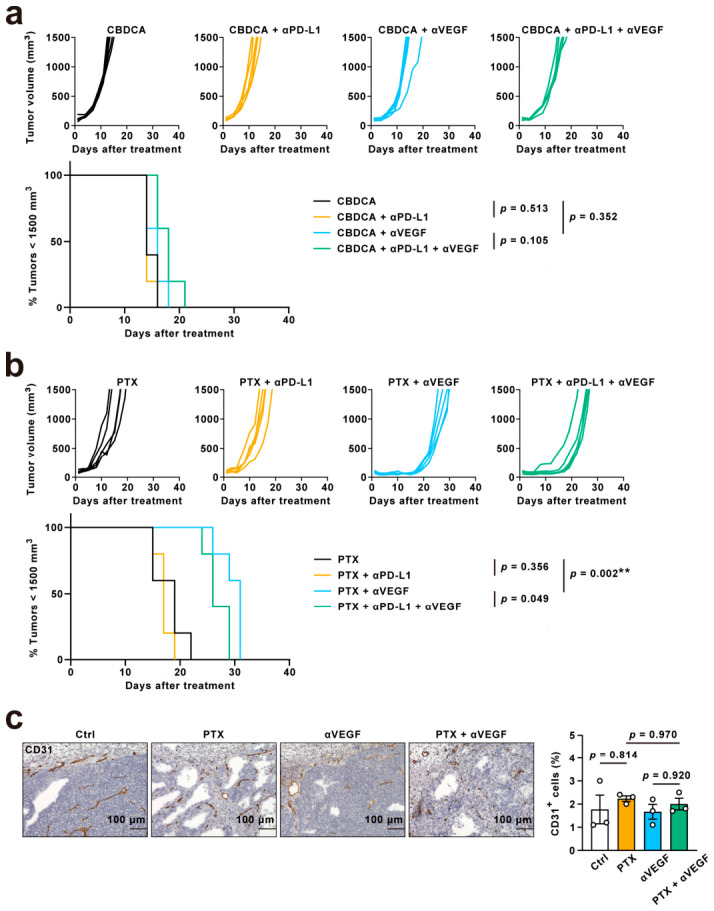
Antitumor activity of paclitaxel combined with anti-PD-L1 and/or low-dose anti-VEGF. (**a**,**b**) Individual tumor growth curves (**top**) and survival curves (**bottom**) for *Egfr*-mutant syngeneic mouse models treated with carboplatin (**a**) or paclitaxel (**b**) in combination with anti-PD-L1, low-dose anti-VEGF, or both. The Kaplan–Meier plot shows the percentage of animals with tumor volumes of less than 1500 mm^3^. *n* = 5 mice per group. (**c**) Anti-angiogenic effect of paclitaxel and low-dose anti-VEGF in *Egfr*-mutant tumors. Representative images of immunohistochemistry for CD31 staining in *Egfr*-mutant lung tumors treated with paclitaxel, low-dose anti-VEGF, or the combination (**left**) and quantification of the percentage area of CD31^+^ cells (**right**). *n* = 3 samples per group. Data are presented as mean ± S.E.M.; statistical analyses were performed using the log-rank test (**a**,**b**) or one-way ANOVA with Tukey’s post hoc test (**c**); **, *p* < 0.01.

We evaluated the combination effects of carboplatin or paclitaxel with anti-PD-L1 or anti-VEGF in our syngeneic *Egfr*-mutant lung cancer model. However, neither carboplatin nor paclitaxel, when combined with anti-PD-L1 antibody, showed superior antitumor activity compared to their respective monotherapies (Figure 3a,b). Similarly, combining carboplatin with low-dose anti-VEGF (1 mg/kg) did not significantly enhance the antitumor effect (Figure 3a). In contrast, the combination of paclitaxel with low-dose anti-VEGF significantly suppressed tumor growth compared to either agent alone (Figure 3b). To characterize the vascular and stromal microenvironment, we performed immunohistochemical staining for CD31 and α-smooth muscle actin (αSMA). The CD31^+^ vascular area in tumors treated with paclitaxel, low-dose anti-VEGF, or their combination was not significantly decreased compared with controls (Figure 3c), indicating that the antitumor activity of the combination occurred without significant vascular pruning. In contrast, the proportion of αSMA^+^ cells was significantly increased broadly within the stromal area in tumors treated with the combination of paclitaxel and low-dose anti-VEGF, but not in those treated with either monotherapy (Supplementary Figure S4), indicating a combination-induced stromal alteration.

### 3.4. Limited Role of Cd8^+^ T-Cell-Mediated Immunity in the Antitumor Effect of Paclitaxel and Low-Dose Anti-Vegf

**Figure 4:**
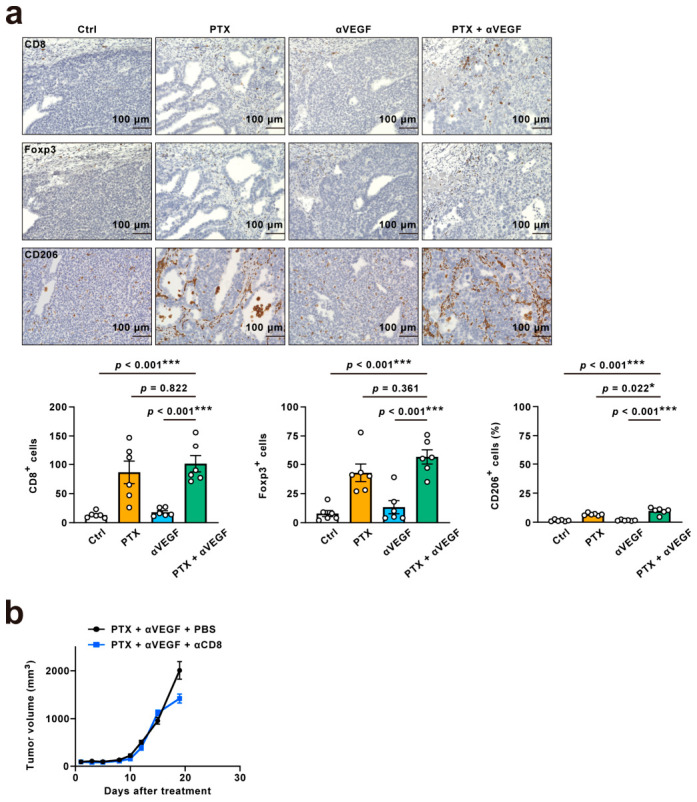
Limited role of CD8^+^ T cells in the antitumor effects of paclitaxel and low-dose anti-VEGF in *Egfr*-mutant tumors in vivo. (**a**) Immunohistochemical evaluation of immune cells infiltrating *Egfr*-mutant lung tumors treated with paclitaxel, low-dose anti-VEGF, or the combination (**top**: representative images, **bottom**: quantification of positive cells). *n* = 6 samples per group. (**b**) Mean tumor volumes in CD8^+^ T cell-depleted *Egfr*-mutant lung tumor-bearing immunocompetent mice treated with paclitaxel and low-dose anti-VEGF. *n* = 6 mice per group. Data are presented as mean ± S.E.M.; statistical analyses were performed using one-way ANOVA with Tukey’s post hoc test; *, *p* < 0.05; ***, *p* < 0.001.

To determine whether the enhanced antitumor activity of paclitaxel and low-dose anti-VEGF was mediated through CD8^+^ T-cell immunity, we next assessed immune cell populations in the TME, including CD8^+^ T cells, Foxp3^+^ Treg cells, and CD206^+^ M2-like macrophages. Although the combination significantly increased CD8^+^ cell infiltration compared to control, no additional increase was observed compared to paclitaxel monotherapy (Figure 4a and Figure S5). Anti-VEGF antibody is reported to have the potential to inhibit Treg cells or M2 macrophages; while Foxp3^+^ cells showed no significant change compared to paclitaxel alone, CD206^+^ cells showed a statistically significant but numerically modest increase in the combination group compared to paclitaxel alone (Figure 4a). Adding anti-PD-L1 to the combination of paclitaxel and low-dose anti-VEGF did not significantly enhance the antitumor effect, a result similar to the lack of effect observed with the combination of carboplatin, anti-VEGF (Figure 3a,b). Administration of the anti-CD8α antibody, which resulted in persistent depletion of splenic CD8^+^ T cells (>90% across all time points; Supplementary Figure S6), did not attenuate the antitumor effect of the combination (Figure 4b). These findings indicate that the antitumor effect of the combination of paclitaxel and anti-VEGF is mediated through a CD8^+^ T-cell-independent mechanism and is insufficient to sensitize *Egfr*-mutant lung tumors with non-inflamed TME to anti-PD-L1.

### 3.5. Paclitaxel and Low-Dose Anti-Vegf Antitumor Activity Is Accompanied by Nk Cell Infiltration

**Figure 5:**
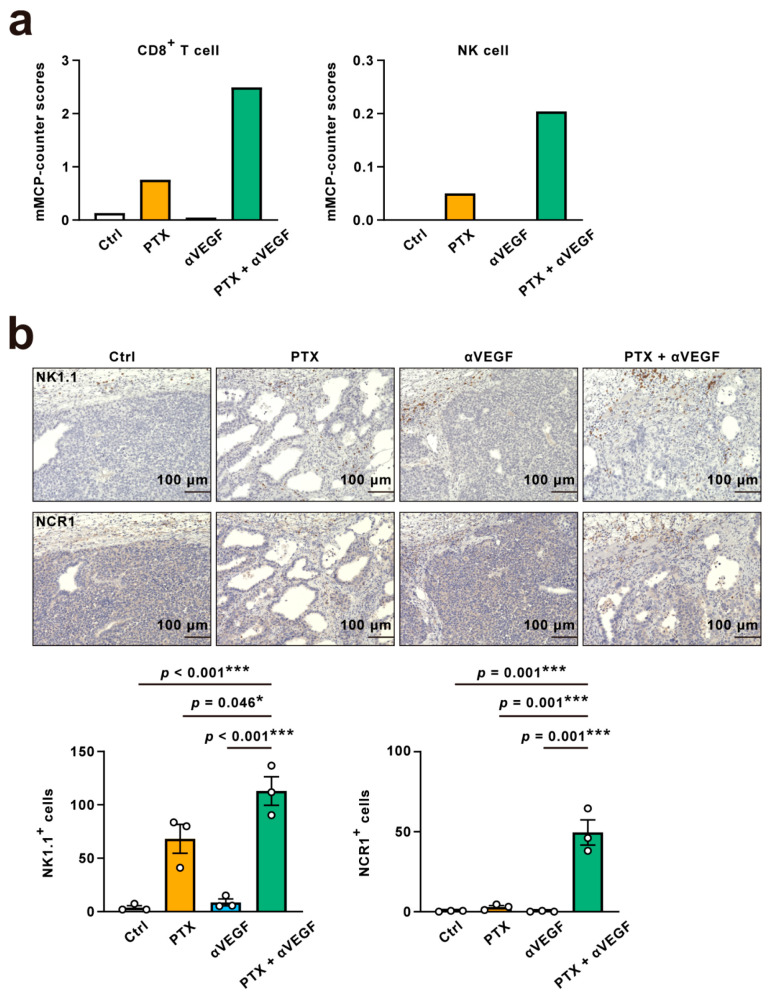
Increased NK cells in *Egfr*-mutant tumors treated with paclitaxel and low-dose anti-VEGF. (**a**) Estimation of tumor-infiltrating immune cell proportion using the mMCP-counter algorithm from bulk RNA sequencing data of *Egfr*-mutant lung tumors treated with paclitaxel, low-dose anti-VEGF, or the combination. *n* = 2 samples per group. (**b**) Immunohistochemical evaluation of NK1.1^+^ cells and NCR1^+^ cells infiltrating *Egfr*-mutant lung tumors treated with paclitaxel, low-dose anti-VEGF, or the combination (**top**: representative images; **bottom**: quantification of positive cell counts). *n* = 3 samples per group. Data are presented as mean (**a**), or mean ± S.E.M. (**b**); statistical analyses were performed using one-way ANOVA with Tukey’s post hoc test; *, *p* < 0.05; ***, *p* < 0.001.

To explore the immune factors contributing to the antitumor effect of the combination of paclitaxel and low-dose anti-VEGF, we performed RNA sequencing in our syngeneic model. Tumors were harvested from mice treated with vehicle, paclitaxel alone, low-dose anti-VEGF, or the combination. To estimate the immune composition within the tumors, we performed mMCP-counter method analysis using the RNA-seq data. This analysis revealed a trend toward an increase in the expression of genes related to not only CD8^+^ T cells but also NK cells in the tumors treated with the combination therapy compared with those treated with paclitaxel or anti-VEGF antibody alone (Figure 5a). To confirm this finding, IHC demonstrated a significant increase in NK1.1^+^ cells in the tumors treated with the combination compared to all other groups, including paclitaxel monotherapy or anti-VEGF monotherapy, and vehicle control (Figure 5b). To further characterize the infiltrating NK cells, we additionally examined NCR1 (NKp46), an activating receptor associated with natural cytotoxicity. NCR1^+^ cells were similarly increased in the combination group (Figure 5b), suggesting that the infiltrating NK cells possess natural cytotoxic potential. These findings raise the possibility that NK cells are a candidate effector population contributing to the antitumor effect of the combination, warranting further investigation.

## 4. Discussion

While anti-VEGF/VEGFR therapies with cytotoxic chemotherapy have been approved for advanced NSCLC as anti-angiogenic agents, the U.S. Food and Drug Administration has not approved the quadruple therapy (Chemo-IO plus bevacizumab) for *EGFR*-mutant lung cancers. In contrast, the European Medicines Agency and the Japan Pharmaceuticals and Medical Devices Agency have approved this quadruple therapy for the *EGFR*-mutant subset following EGFR-TKI failure [29,30,31]. This regulatory disparity underscores the critical need to determine whether anti-VEGF antibody enhances antitumor immunity in this clinical setting. To address this question without the confounding influence of prior TKI-induced TME alterations, we employed an EGFR-TKI-naïve syngeneic model. In this model, we demonstrated that anti-VEGF antibody did not sensitize *Egfr*-mutant tumors to ICI or Chemo-IO. Thus, while anti-VEGF therapy inhibited tumor growth, our findings suggest that its potential to modulate the non-inflamed TME or sensitize tumors to ICIs is limited in EGFR-TKI-naïve tumors with a non-inflamed TME.

The inconsistent clinical outcomes of quadruple therapy may be attributed to the heterogeneity of the TME of *EGFR*-mutant tumors [15,16,17,32]. For example, a biomarker study of the ATTLAS trial showed that a subset of patients with high inflammatory scores (tumor-infiltrating lymphocytes ≥ 20%) derived significant progression-free survival benefits from the quadruple combination therapy [33]. However, these features are not representative of the major *EGFR*-mutant population, which typically exhibits a non-inflamed TME and low PD-L1 expression [6]. Our EGFR-TKI-naïve mouse model recapitulates this predominant phenotype of *EGFR*-mutant lung cancer, specifically exhibiting a non-inflamed TME and low PD-L1 expression [21]. Therefore, while our model did not show a clear benefit from the combination of anti-VEGF and anti-PD-L1 antibodies, the combination therapy may produce antitumor immunity in *Egfr*-mutant tumors with immunologically inflamed features. Our study underscores the importance of developing predictive biomarkers to identify patients in whom the quadruple combination therapy could sensitize tumors to ICIs in *EGFR*-mutant lung cancer.

Previous studies revealed that some cytotoxic chemotherapeutic agents such as taxanes possess immune-related mechanisms that contribute to their antitumor activity, but others such as cisplatin do not [34]. Multiple preclinical studies showed that paclitaxel has an immunomodulatory effect, including activation of CD8^+^ T cells, or NK cells in breast cancer or melanoma models [35,36]. However, no preclinical study has evaluated the role of paclitaxel as an immunomodulator in *Egfr*-mutant lung cancer models using syngeneic mouse models. In the present study, we demonstrated that paclitaxel, unlike carboplatin, increased intratumoral CD8^+^ T-cell infiltration in our syngeneic *Egfr*-mutant lung cancer model, without a concomitant upregulation of PD-1, a marker that can reflect both recent T-cell receptor engagement and chronic exhaustion [28]. Based on this increased infiltration and the superior antitumor effect of combination therapy with paclitaxel and anti-VEGF, we initially hypothesized that the anti-VEGF therapy might augment the CD8^+^ T cell-mediated antitumor activity of paclitaxel. Contrary to our hypothesis, neither the addition of an anti-PD-L1 antibody nor CD8^+^ T cell depletion altered the therapeutic effect. These functional data, together with the comparable CD8^+^/Foxp3^+^ ratio and the lack of PD-1 upregulation, suggest that the paclitaxel-induced lymphocyte infiltration represents a non-selective lymphocyte recruitment rather than a selective accumulation of tumor-reactive effector CD8^+^ T cells. Thus, a CD8^+^ T-cell-mediated immune response does not contribute to the enhanced antitumor effect induced by the combination of paclitaxel and anti-VEGF. In parallel, immunohistochemical analysis revealed that the combination preserved CD31^+^ vascular density while increasing αSMA^+^ cells broadly within the stromal area. Classical structural vascular normalization typically requires both reduced microvessel density and increased pericyte coverage [37]; although single-marker αSMA staining precludes definitive classification of the expanded cell population, the absence of microvessel pruning indicates that the canonical pattern of vascular normalization was not evident, and the findings are instead consistent with a combination-induced stromal alteration. RNA sequencing and immunohistochemistry revealed an increase in NK cell infiltration in tumors treated with the combination, raising the possibility that innate immune cells beyond CD8^+^ T cells may contribute to the antitumor effects, warranting further investigation in *EGFR*-mutant lung cancer.

This study has several limitations. Our findings are based on a syngeneic mouse model that recapitulates human lung cancer harboring an *Egfr* ex19del mutation with a non-inflamed TME; however, we have not evaluated our findings in other types of *EGFR* mutations or co-mutations. Furthermore, unlike the patients enrolled in clinical trials of quadruple therapy, our model used tumors that were not pre-treated with EGFR-TKIs, which may affect the TME and the sensitivity to ICIs or anti-VEGF therapy. Our characterization of vascular and stromal changes was limited by the use of single-marker αSMA immunohistochemistry, which cannot definitively distinguish pericytes from other stromal components, and by the absence of direct physiological readouts of vascular function such as intratumoral hypoxia. The immunophenotypic characterization of CD8^+^ T cells was based on PD-1 expression alone; co-staining with additional activation markers such as Ki-67 or CD69 would have allowed a more comprehensive assessment of their functional state. In addition, although systemic depletion of CD8^+^ T cells (>90% reduction) was confirmed in the spleen (Supplementary Figure S6), the depletion efficiency within the tumor was not directly assessed in the present cohort. Thus, this finding should be interpreted with appropriate caution. Due to the difficulty of obtaining clinical samples, we were also unable to validate our findings in a clinical setting, which reduces their direct clinical relevance. Moreover, this study evaluated a single VEGF-A-targeted antibody at two specific doses; the immunomodulatory efficacy of anti-angiogenic therapy can vary with dose [23], treatment duration [22], and the type of inhibitor used, and therefore alternative anti-VEGF/VEGFR strategies could yield different immunological outcomes in *Egfr*-mutant tumors. Our results should therefore be interpreted with caution when extrapolating to human lung cancer, and the roles of NK cells and other innate immune populations in mediating the antitumor effects of the combination remains to be elucidated. Future studies should also explore combinations with modalities that elicit immunogenic cell death, such as radiotherapy [34,38], to further convert the non-inflamed TME of *Egfr*-mutant tumors into an immunologically active state.

## 5. Conclusions

Our study reveals the limited role of the evaluated anti-VEGF-A antibody in activating antitumor immunity in combination with anti-PD-L1, either alone or with paclitaxel, in EGFR-TKI-naïve *Egfr*-mutant tumors with a non-inflamed TME. The combination of paclitaxel and low-dose anti-VEGF suppressed tumor growth through a CD8^+^ T-cell-independent mechanism, suggesting that immunotherapeutic strategies beyond CD8^+^ T-cell-mediated immunity may warrant further investigation in this challenging disease subset.

## Data Availability

The RNA-sequencing data presented in this study are openly available in the GEO database, reference number (GSE330658). Other raw data, including in vivo tumor growth and IHC data, are contained within the article and its Supplementary Materials.

## Supplementary Materials

The following supporting information can be downloaded at https://www.mdpi.com/article/10.3390/curroncol33060315/s1, Supplementary Figure S1: Efficacy of targeted therapies in *Egfr*-mutant lung tumors and MC38 colon adenocarcinoma; Supplementary Figure S2: Tumor microenvironment of *Egfr*-mutant tumors treated with anti-PD-L1 and/or low-dose anti-VEGF; Supplementary Figure S3: Gating strategies for flow cytometric analysis of tumor-infiltrating lymphocytes; Supplementary Figure S4: Effect of paclitaxel and low-dose anti-VEGF on stromal αSMA expression in *Egfr*-mutant tumors; Supplementary Figure S5: Flow cytometric evaluation of CD8^+^ T cells infiltrating *Egfr*-mutant lung tumors treated with paclitaxel and low-dose anti-VEGF; Supplementary Figure S6: Flow cytometric validation of CD8^+^ T-cell depletion in the spleen.

## Abbreviations

The following abbreviations are used in this manuscript:

PD-L1	Programmed death-ligand 1
PD-1	Programmed cell death-1
VEGF	Vascular endothelial growth factor
VEGFR	VEGF receptor
EGFR	Epidermal growth factor receptor
TKIs	Tyrosine kinase inhibitors
ICIs	Immune checkpoint inhibitors
TME	Tumor microenvironment
TMB	Tumor mutational burden
IHC	Immunohistochemistry
FCM	Flow cytometry
NK	Natural killer
NSCLCs	Non-small cell lung cancers
Tregs	Regulatory T-cells
CTL	Cytotoxic T lymphocytes
TILs	Tumor-infiltrating lymphocytes
Chemo-IO	Chemotherapy plus ICI
GEMM	Genetically engineered mouse model
PBS	Phosphate-buffered saline
RNA-seq	RNA-sequencing
TPM	Transcripts per million
RPK	Reads per kilobase
mMCP	Murine Microenvironment Cell Population
NCR1	Natural cytotoxicity triggering receptor 1
αSMA	Alpha smooth muscle actin
