# Supplementary material for: Limited Immune-Mediated Efficacy of Anti-PD-L1/VEGF in EGFR-TKI-Naïve Egfr-Mutant Lung Cancer with Non-Inflamed Tumor Microenvironment
Source: Curr Oncol. 2026 May 27;33(6):315. doi: 10.3390/curroncol33060315 (PMC13297713; doi:10.3390/curroncol33060315)
Supplement: Supplementary file 1 [file curroncol-33-00315-s001.zip › curroncol-4277560-supplementary.pdf]

Suppl. Fig. S1

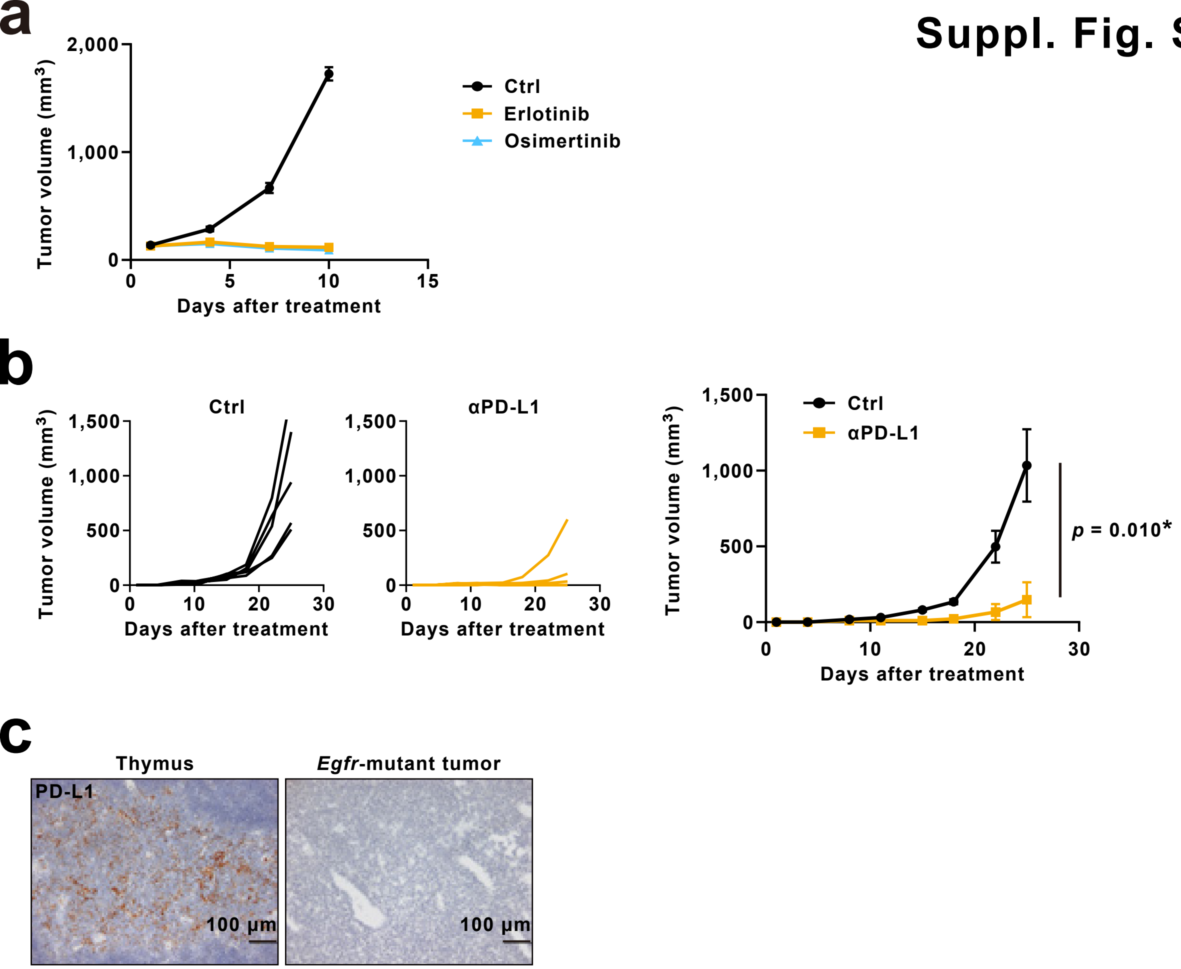

Supplementary Fig. S1 Efficacy of targeted therapies in *Egfr*-mutant lung tumors and MC38 colon adenocarcinoma. a. Antitumor effects of erlotinib or osimertinib in *Egfr*-mutant mouse model. n = 5 mice per group. **b.** Antitumor effects of anti-PD-L1 in MC38 tumor-bearing mice. n = 5 mice per group. **c.** Intratumoral PD-L1 expression in an *Egfr*-mutant lung tumors assessed by immunohistochemistry. Thymus was used as a positive control. Data are presented as mean ± S.E.M.; statistical analyses were performed using Student's t-test; *, $p < 0.05$. Ctrl, control; αPD-L1, anti-PD-L1 antibody.

Suppl. Fig. S2

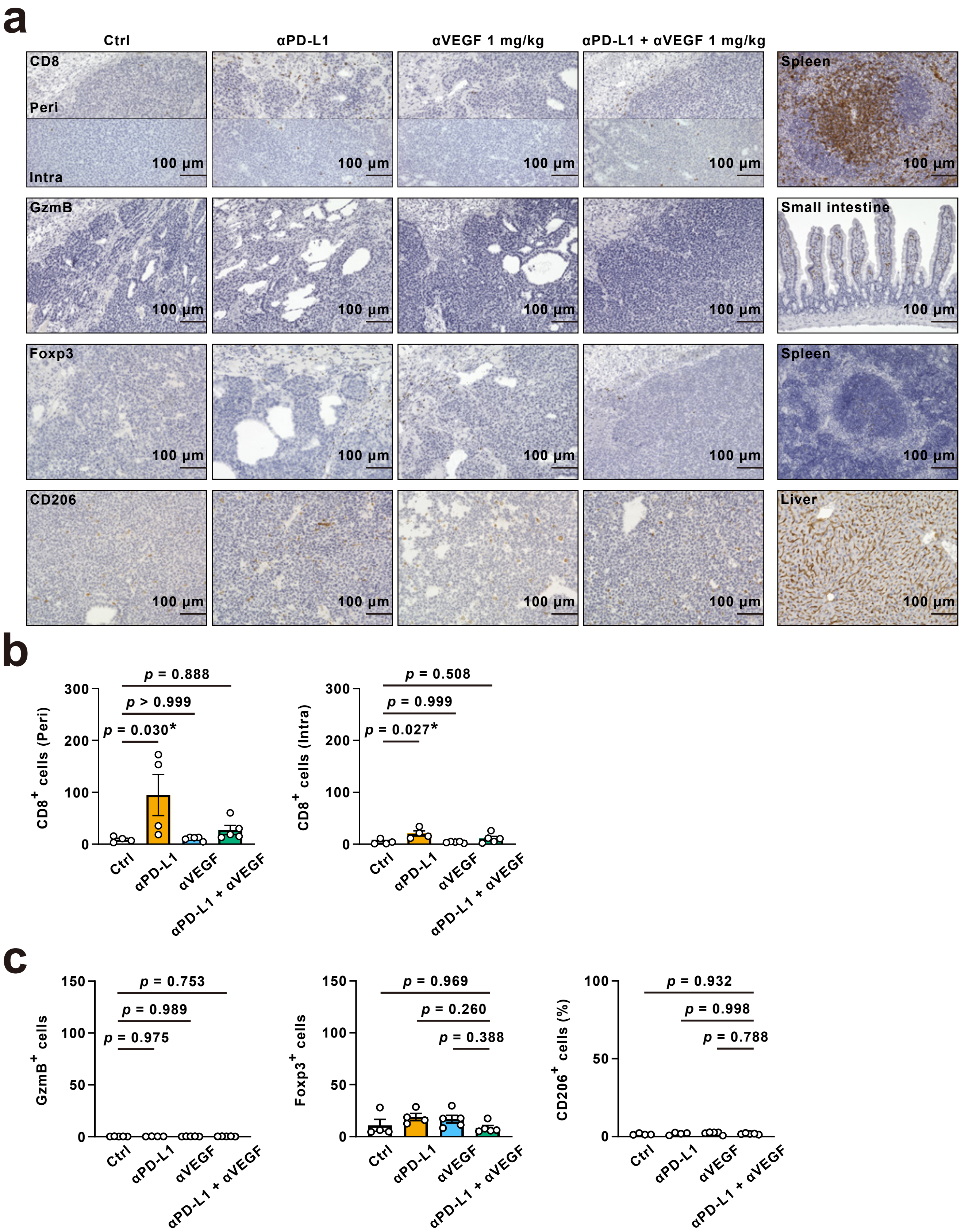

Supplementary Fig. S2 Tumor microenvironment of *Egfr*-mutant tumors treated with anti-PD-L1 and/or low-dose anti-VEGF. a. Immunohistochemical evaluation of immune cells infiltrating *Egfr*-mutant lung tumors treated with anti-PD-L1, low-dose anti-VEGF, or the combination. Positive controls for the antibodies used in each immunohistochemical analysis are shown in the right column (spleen for CD8 and Foxp3, small intestine for GzmB, and liver for CD206). For CD8 staining, the images are shown separately for the peritumoral (Peri) and intratumoral (Intra) areas. **b.** Quantification of $CD8^+$ cells in the peritumoral (left) and intratumoral (right) areas. **c.** Quantification of GzmB-, Foxp3-, and CD206-positive cells in the immunohistochemical images in a. n = 4–5 samples per group. Data are presented as mean ± S.E.M.; statistical analyses were performed using one-way ANOVA with Tukey's post hoc test; *, $p < 0.05$. Ctrl, control; αPD-L1, anti-PD-L1 antibody; αVEGF, anti-VEGF antibody.

Suppl. Fig. S3

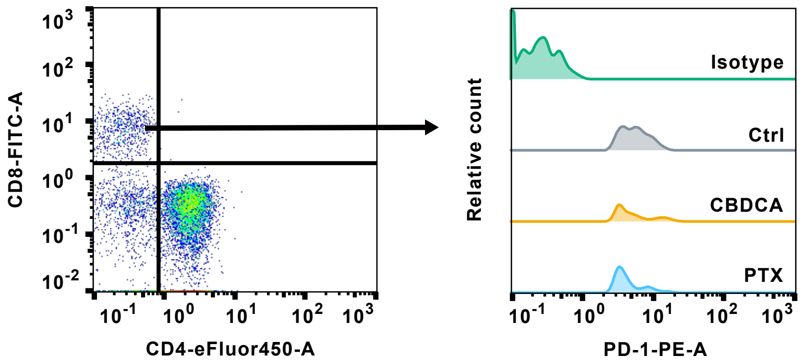

Supplementary Fig. S3 Gating strategies for flow cytometric analysis of tumor-infiltrating lymphocytes. Representative gating strategies for $CD8^+$ cells (left) and histogram plots comparing the fluorescence intensity of PD-1 on $CD8^+$ cells (right) in tumors treated with carboplatin or paclitaxel. Ctrl, control; CBDCA, carboplatin; PTX, paclitaxel.

Suppl. Fig. S4

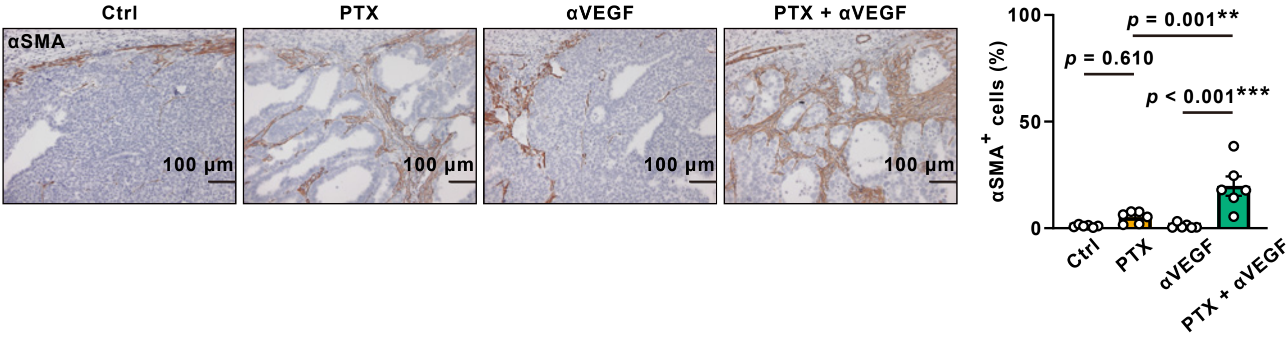

Supplementary Fig. S4 Effect of paclitaxel and low-dose anti-VEGF on stromal αSMA expression in *Egfr*-mutant tumors. Immunohistochemical analysis of αSMA⁺ cells in tumors treated with paclitaxel, low-dose anti-VEGF, or the combination (left: representative images; right: quantification of positive cells). n = 6 samples per group. Data are presented as mean ± S.E.M.; statistical analyses were performed using one-way ANOVA with Tukey's post hoc test; **, $p < 0.01$; ***, $p < 0.001$. Ctrl, control; PTX, paclitaxel; αVEGF, anti-VEGF antibody.

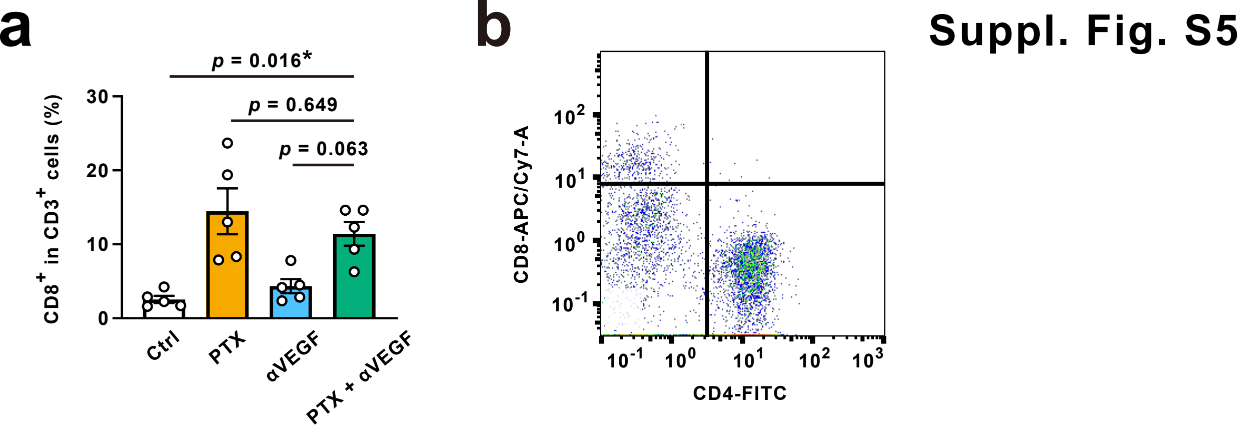

Supplementary Fig. S5 Flow cytometric evaluation of CD8⁺ T cells infiltrating *Egfr*-mutant lung tumors treated with paclitaxel and low-dose anti-VEGF. a. Ratio of CD8⁺ cells among CD3⁺ cells in dissociated tumors treated with paclitaxel and low-dose anti-VEGF. n = 5 samples per group. **b.** Representative gating strategies for CD8⁺ cells. Data are presented as mean ± S.E.M; statistical analyses were performed using one-way ANOVA with Tukey's post hoc test.; *, $p < 0.05$. Ctrl, control; CBDCA, carboplatin; PTX, paclitaxel; αVEGF, anti-VEGF antibody.

Suppl. Fig. S6

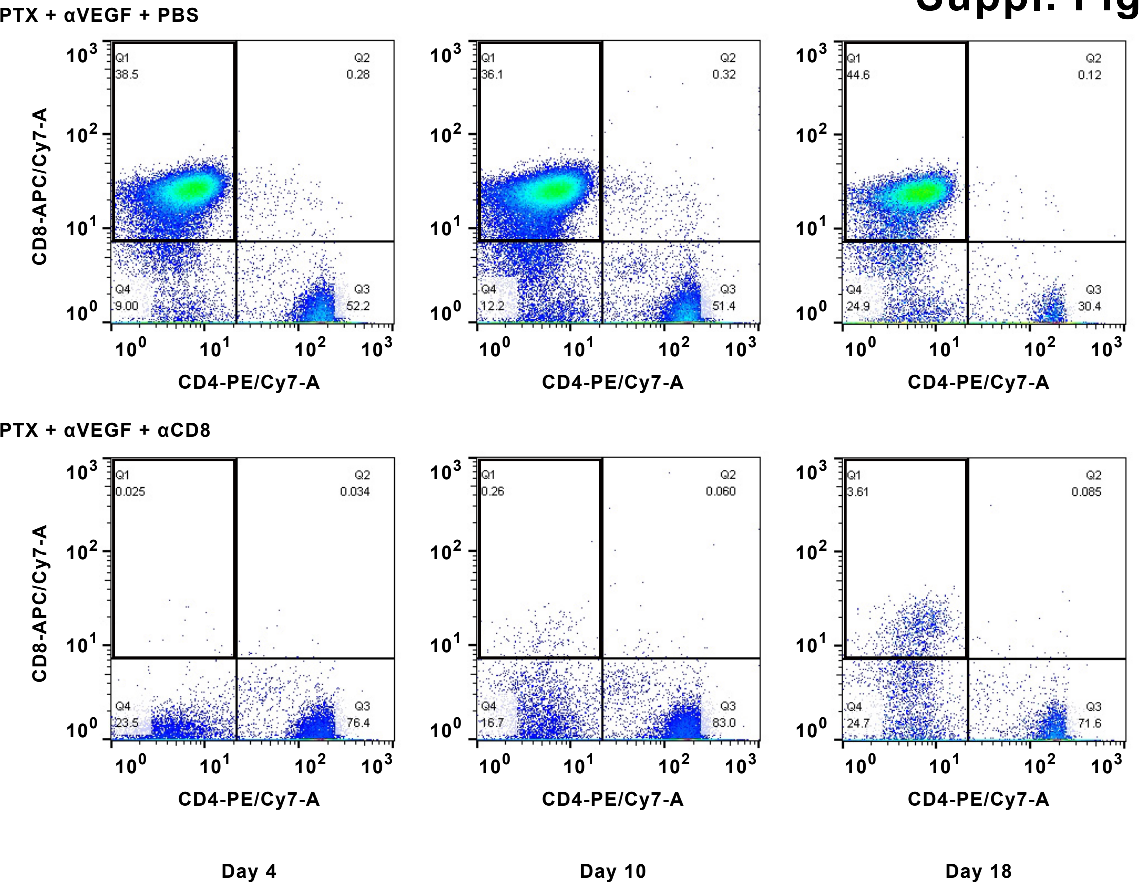

Supplementary Fig. S6 Flow cytometric validation of $CD8^+$ T-cell depletion in the spleen. Splenocytes were harvested from *Egfr*-mutant tumor-bearing mice treated with paclitaxel (20 mg/kg) plus low-dose anti-VEGF (1 mg/kg) and either PBS or anti-CD8α antibody (10 mg/kg) on days 4, 10, and 18 after the start of antibody administration. Representative flow cytometry plots show $CD8^+$ T cells within the lymphocyte gate.
